# Endoscopic ultrasound-guided hepaticogastrostomy with bridging as reintervention for stent occlusion in malignant hilar biliary obstruction

**DOI:** 10.1055/a-2186-4941

**Published:** 2023-11-22

**Authors:** Fumitaka Niiya, Hirotoshi Ishiwatari, Junya Sato, Hiroyuki Matsubayashi, Hiroyuki Ono

**Affiliations:** 1 Endoscopy, Shizuoka Cancer Center, Sunto-gun, Shizuoka; 2 Internal Medicine, Showa University Fujigaoka Hospital, Yokohama

Placement of multiple metal stents by endoscopic retrograde cholangiopancreatography for malignant hilar biliary obstruction (MHBO) contributes to longer stent patency 1 2 . However, endoscopic reintervention is technically difficult when stents are occluded 3 . Endoscopic ultrasonography-guided hepaticogastrostomy (EUS-HGS) is performed for the drainage of the left hepatic bile duct in MHBO. A bridging technique during EUS-HGS can be a promising method for treating isolated right hepatic bile duct obstruction 4 5 . However, the bridging procedure has yet to be performed as a reintervention for obstructed metal stents in MHBO.

A 58-year-old woman with gallbladder carcinoma and a history of multiple endoscopic treatments for MHBO, including placement of five stents, was admitted with cholangitis ( Fig. 1 a ). Computed tomography revealed dilatation of the B2 intrahepatic bile duct and right posterior bile duct (RPD). We failed to insert a stent into the RPD using the endoscopic transpapillary approach. Moreover, EUS-guided RPD drainage from the duodenum was impossible because tumor obstruction prevented the puncture of the RPD. Therefore, EUS-HGS was performed using the bridging method ( Video 1 ).

**Fig. 1:**
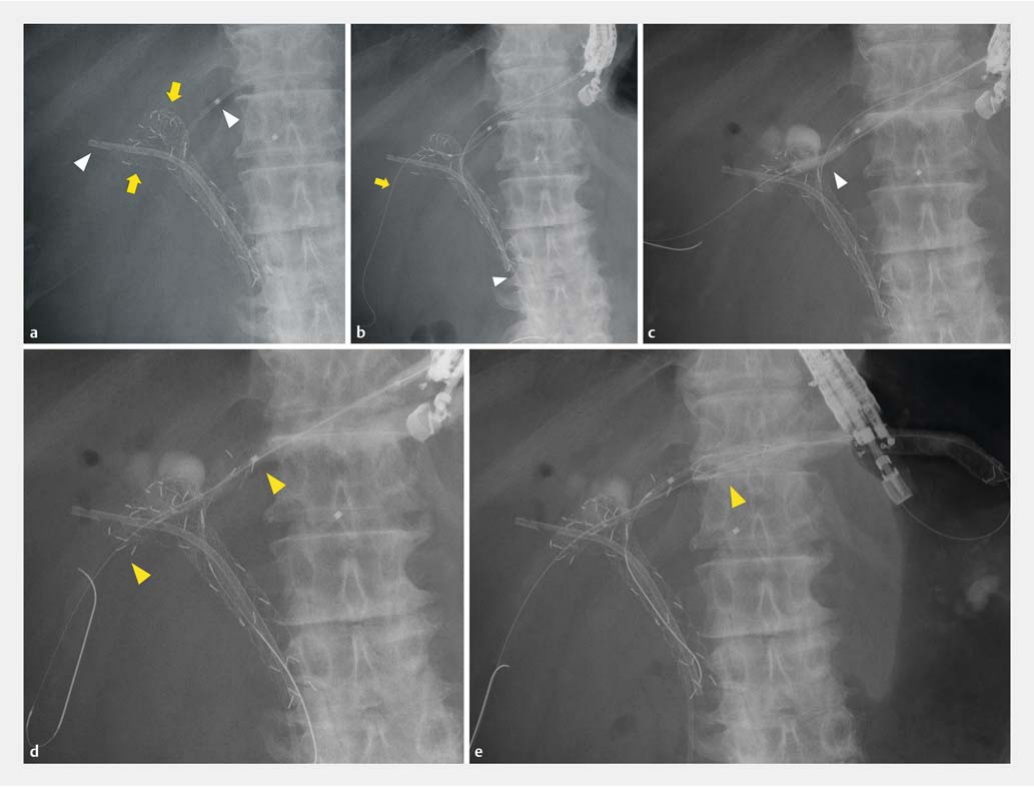
Fluoroscopic images of endoscopic ultrasound-guided hepaticogastrostomy with bridging. **a** Biliary stents on admission. Over three separate sessions of endoscopic treatment by endoscopic retrograde cholangiopancreatography, three metal stents had been placed in the anterior and posterior bile duct by a partial stent-in-stent technique (arrows), followed by placement of one plastic stent into the anterior bile duct and another plastic stent into the B3 intrahepatic bile duct (arrowheads). **b** After puncture of the B2 intrahepatic bile duct from the stomach under endoscopic ultrasound guidance, a guidewire was inserted into the bile duct and the duodenum (arrowhead). Then, a 0.025-inch hydrophilic guidewire was advanced into the right posterior bile duct through the mesh of the previously placed metal stent (arrow) using a double-lumen cannula. **c** Balloon dilation was performed to dilate the mesh of the previously placed metal stents using a thin-tipped balloon catheter to facilitate subsequent placement of a self-expandable metal stent (arrowhead). **d** An uncovered self-expandable metal stent was inserted through the mesh of the previously placed metal stents, between the right posterior bile duct and the left hepatic bile duct (arrowheads). **e** A partially covered self-expandable metal stent was placed from the left hepatic bile duct to the stomach (arrowhead).

**Video 1** We successfully performed biliary drainage of the posterior intrahepatic bile duct using a bridging method from the endoscopic ultrasound-guided transgastric approach in a patient after placing multiple metal stents.


Following the puncture of B2 under EUS guidance, a guidewire was advanced beyond the MHBO and into the duodenum. Subsequently, a double-lumen cannula (Uneven double-lumen cannula; Piolax Medical Devices, Inc., Tokyo, Japan) was inserted, followed by a 0.025-inch hydrophilic guidewire into the RPD through the mesh of the previously placed metal stent ( Fig. 1 b ). After dilating the mesh of the metal stent using a thin-tipped balloon catheter with a diameter of 4 mm (REN Balloon Dilation Catheter; Kaneka Corporation, Osaka, Japan) ( Fig. 1 c ), an uncovered self-expandable metal stent (Niti-S biliary; Tae-Woong Medical, Seoul, Korea) was inserted between the RPD and left hepatic bile duct ( Fig. 1 d ). Finally, we placed a partially covered, self-expandable metal stent from the left hepatic bile duct to the stomach ( Fig. 1 e ).

Recovery was uneventful, and cholangitis subsided within a few days. Although this patient had multiple metal stents placed, EUS-HGS with the bridging method was a feasible treatment option.

Endoscopy_UCTN_Code_TTT_1AS_2AD

## Notes

Endoscopy E-Videos

https://eref.thieme.de/e-videos
*E-Videos* is an open access online section of the journal *Endoscopy* , reporting on interesting cases and new techniques in gastroenterological endoscopy. All papers include a high-quality video and are published with a Creative Commons CC-BY license. Endoscopy E-Videos qualify for HINARI discounts and waivers and eligibility is automatically checked during the submission process. We grant 100% waivers to articles whose corresponding authors are based in Group A countries and 50% waivers to those who are based in Group B countries as classified by Research4Life (see: https://www.research4life.org/access/eligibility/ ). This section has its own submission website at https://mc.manuscriptcentral.com/e-videos
